# Washington State Homœopathic Medical Society

**Published:** 1894-06

**Authors:** 


### WASHINGTON STATE HOMŒOPATHIC MEDICAL SOCIETY.

The fifth annual session of the Washington State Homœopathic Medical Society was held in Tacoma on May 8th.

The meeting was full of interest, the chief business of the day being the reading of valuable papers by various members.

Considerable business was transacted in executive session, matters of interest only to the society being treated. During the day the following resolution was unanimously adopted:

"*Resolved*, That we most earnestly indorse and approve the efforts being made to secure the meeting of the American Institute of Homœopathy at some point on the Pacific coast, in 1896, and that we pledge our efforts as individuals and as a society to make such a meeting a success both in numbers and attendance."

The papers read were by Dr. T. M. Young, of Seattle, on "Placenta Prævia;" by Dr. T. W. Southworth, of Tacoma, on "The Limits of Therapeutics;" by Dr. W. W. Misner, of

Tacoma, on "Apoplexy;" by Dr. Sarah Kendall, of Seattle, on "Puerperal Sepsis and Intra-uterine Irrigation;" by Dr. E. Weldon Young, of Seattle, on "Neurasthenia;" Dr. T. R. Hall, of Tacoma, on the "Perils of the New Born;" by Dr. F. A. Churchill, of Seattle, on "Electricity and the Disorders of Menstruation;" Dr. H. B. Bagley, of Seattle, on "Potency;" Dr. E. D. Olmstead, of Spokane, on "Sanitation;" Dr. C. M. Baldwin, of Port Townsend, "Climatic Influences in Heart and Lung Troubles;" Dr. R. C. Corey, of Olympia, "A Clinical Case;" Dr. C. A. Walsh, of Seattle, "A Four Years' Record, or Over Forty Cases of Successful Abdominal Section;" Dr. T. F. Thompson, of Snohomish, "A Verification of an Arnica Keynote;" Dr. E. D. Olmstead, of Spokane, a statistical paper, "Figures Won't Lie."

Dr. Munson, of Tacoma, president of the society, delivered a very able annual address. In it he devoted special attention to the subject of medical legislation. He referred to the advanced position taken by the homœopathic school on all matters calculated to elevate the standard of the medical profession, and urged the members of the society to do all that they could to assist in the work.

Among the most interesting and scientific papers read at the session was one on apoplexy by Dr. W. W. Misner, of this city. The doctor treated the subject in its various phases in a most thorough and exhaustive manner. He took up the etiology, symptomatology, diagnosis, and treatment, and handled them creditably.

The paper that probably attracted more attention, and was remarkable for the research displayed, was that by Dr. E. Weldon Young, of Seattle, whose subject was "Neurasthenia," the American disease from overwork.

The old officers were: President, Dr. Clinton Munson, of Tacoma; Vice-President, Dr. F. A. Churchill, of Seattle; Secretary, Dr. E. Weldon Young, of Seattle; Treasurer, Dr. Sarah Kendall, of Seattle; Board of Censors, Dr. T. M. Young, of Seattle; Dr. F. R. Hill, of Tacoma; Dr. E. F. Stevens, of Seattle; Dr. Sarah Kendall, of Seattle.—*Tacoma Ledger*.

